# Cooperative Virus-Virus Interactions: An Evolutionary Perspective

**DOI:** 10.34133/2022/9819272

**Published:** 2022-08-09

**Authors:** Ernesto Segredo-Otero, Rafael Sanjuán

**Affiliations:** Institute for Integrative Systems Biology (I2SysBio), Consejo Superior de Investigaciones Científicas-Universitat de València, C/ Catedrático Agustín Escardino 9, 46980 Paterna, València, Spain

## Abstract

Despite extensive evidence of virus-virus interactions, not much is known about their biological significance. Importantly, virus-virus interactions could have evolved as a form of cooperation or simply be a by-product of other processes. Here, we review and discuss different types of virus-virus interactions from the point of view of social evolution, which provides a well-established framework for interpreting the fitness costs and benefits of such traits. We also classify interactions according to their mechanisms of action and speculate on their evolutionary implications. As in any other biological system, the evolutionary stability of viral cooperation critically requires cheaters to be excluded from cooperative interactions. We discuss how cheater viruses exploit cooperative traits and how viral populations are able to counteract this maladaptive process.

## 1. Introduction

Viruses exhibit a wide range of direct and host-mediated interactions, some of which have been known for decades. Most of these interactions take place in cells coinfected with different viral variants or species. For instance, genetic complementation occurs when two or more viral mutants carrying deleterious mutations at different loci share their gene products to compensate for these defects, thus restoring normal functions [1, 2]. Another well-known example is pseudotyping, which takes place when two different viruses coinfect a cell and produce virions carrying the genome from one virus but some structural proteins from the other virus [3–7]. Two additional examples are provided by embedded viruses, which are retroviruses that fully integrate their genome into the genome of another virus [8–10], and by heterologous transactivation, which occurs when a virus expresses transcription factors that activate promoters of another virus [11, 12]. Negative interactions are also common among coinfecting viruses. In addition to the obvious phenomenon of direct competition for host resources, viruses exhibit mechanisms to inhibit foreign infections, with superinfection exclusion (SIE), a mechanism by which a virus that is infecting a host is able to block other infections, being particularly important [13–17]. Some plant viruses are also able to promote host-wide protection, a process called cross-protection [18, 19].

Viruses can also establish more indirect interactions, which are determined by the characteristics of the environment or the host, particularly the immune system. There are many examples of environmental and immunological interactions between different viruses, some of the most important being altering host susceptibility, modifying or suppressing interferon (IFN) response, or altering immune cell activation [20]. For instance, human immunodeficiency virus (HIV) is able to increase human cytomegalovirus (HCMV) replication in various tissues as a result of lymphocyte activation [21], and humans infected with herpes simplex virus (HSV) are more susceptible to HIV infections and more contagious due to an increased expression of the receptor CCR5 [22, 23]. Innate immunity can also promote negative interaction between viruses [24, 25]. Cooperative and competitive interactions mediated by cross-reactive innate immunity appear to be particularly frequent among respiratory viruses [26].

Most of these well-known interactions involve viruses from different species. Cases of embedding, transactivation, or pseudotyping or the interactions between HIV and HSV may occur as a by-product of a normal process in the infection cycle of the other virus. Less attention was traditionally paid to cases of interactions between viruses of the same species (with the exception of genetic complementation), in which the ability of a viral particle to achieve a successful infection is promoted or hampered by the presence of other viral particles. However, several research lines have emerged in recent years showing this kind of viral interactions in various processes including evasion of host immunity [24] and regulation of virulence [27]. Inclusive fitness theory is a well-established theoretical framework for studying these interactions, which was initially developed to explain the evolution of altruism in higher organisms but allows viral interactions to be formally analyzed in terms of fitness costs and benefits. For a trait to be considered social, its evolution should be determined, at least partially, by the effect it produces on individuals different from the actor.

## 2. Viral Coinfection Mechanisms That Facilitate Interactions

The most common scenario for virus-virus of interactions takes place when multiple infectious particles are present in the same host cell, that is, when there is a high multiplicity of infection (MOI), defined as the number of viral genomes that initiates an infection. Animal viruses typically produce a large number of infectious particles per infected cell, usually ranging from 100 to 1000 [28–30]. In the simplest form of viral spread, these particles diffuse in a viscous medium [31] until they reach a neighbor cell, producing infection foci that create a high local MOI. However, the amount of genomes that are actually transmitted from cell to cell is variable and depends, among other factors, on adsorption efficiency. Bacteriophages can exhibit extremely high adsorption efficiencies [32, 33] that, together with their environmental ubiquity, should promote coinfection and, in turn, select for mechanisms that regulate coinfection levels.

**Figure 1:**
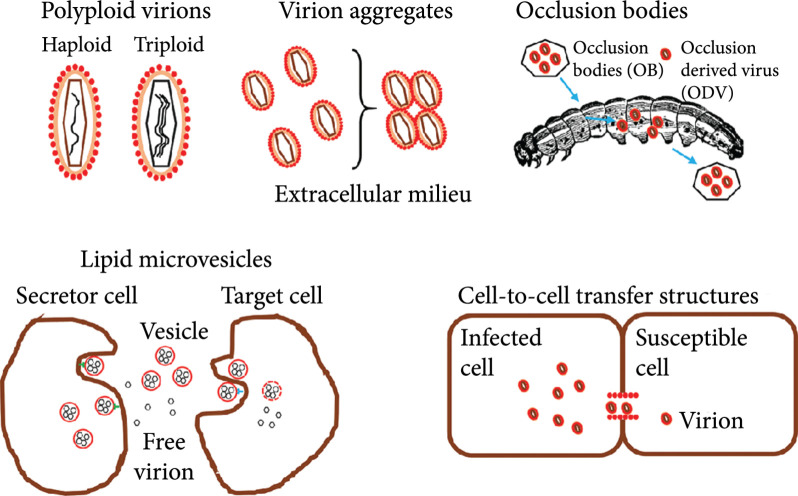
Different types of collective transmission in viruses. Polyploid virions: polyploid virions containing more than one copy of the viral genome. Virion aggregates: aggregates of virions in the extracellular milieu. Occlusion bodies: baculovirus occlusion bodies for interhost transmission. Lipid microvesicles: extracellular vesicles containing multiple virions. Cell-to-cell transfer structures: cell-to-cell transmission mediated by cellular or virus-induced structures that promote group transmission of multiple virions.

A more specialized form of viral spread is cell-to-cell transmission, which should also favor coinfection. Plant viruses can achieve elevated MOIs by delivering multiple viral particles via plasmodesmata [34, 35] (Figure 1). However, the amount of genomes that are transmitted from cell to cell is variable [36, 37]. Animal viruses also exhibit several mechanisms for cell-to-cell transmission such as the formation of actin tails [38], the exploitation of structures like filopodia [39], and tunneling nanotubes [40], as well as the induction of syncytia [41–44] or cell synapses [45, 46].

High MOIs can also be achieved when viral spread occurs through collective infectious units (CIUs, Figure 1). This term describes a wide range of virus-promoted structures that allow cotransmission and delivery of multiple viral genomes in a single host cell [47]. CIUs are found in many different types of viruses, although they have been best characterized in animal viruses. For example, some viruses can form “polyploid” virions containing more than one genome copy [48–52]. CIUs can also be formed when virions aggregate in the extracellular milieu, as has been shown, for instance, for vesicular stomatitis virus (VSV) in saliva [53, 54] or HIV in semen [55, 56]. Occlusion bodies (OBs) of baculoviruses are composed of polyhedrin protein crystals harboring tens of nucleocapsids [57, 58]. A well-described type of CIUs is extracellular vesicles containing multiple viral particles, which have been described in noroviruses [59]; picornaviruses such as coxsackieviruses [60], polioviruses [61], and hepatitis A virus (HAV) [62]; rotaviruses [59]; and marseilleviruses [63]. Despite the great diversity of structures involved in CIU formation, a common theme is the simultaneous delivery of multiple genomes to the same host or cell. CIU-mediated elevation of MOI can be advantageous in different ways, since it could promote cooperative interactions between potentially identical viral genomes (homotypic) or between different genetic variants of a virus (heterotypic).

### 2.1. “Mass” Effects

Everything else being equal, if K virions infect a single cell instead of K different cells, there should be a direct K-fold reduction in the amount of viral progeny produced per cell. For mechanisms promoting coinfection to be beneficial in terms of fitness, they must therefore compensate for this cost by increasing the viral progeny per cell by at least the same factor; or promote some other fitness advantage such as faster infection or greater environmental stability (Table 1). This question has been addressed both theoretically and experimentally, and although there are several possible mechanisms that may confer an evolutionary advantage to CIUs, there is no general answer that applies to all viruses [64].

**Table 1:** Summary of possible fitness advantages of coinfection due to mass effects.

Fitness advantage	Virus	Normalized benefits per capita	Reference
Acceleration of the infection cycle	Poliovirus	Yes	61
	Marseillevirus	No	63
	Influenza A virus	Yes (depending on the MOI and cell type)	70-72
	Human immunodeficiency virus	No	55

Increased per-cell yield	Influenza A virus	Yes (depending on the MOI and cell type)	69-72
	Vaccinia virus	Yes	73
	Vesicular stomatitis virus	Yes	68

Increased infectivity	Infectious bursal disease virus	Yes	51
	Human immunodeficiency virus	No	55
	Vaccinia virus	Yes	73
	Influenza A virus	Depends on segment encapsidation	70

A general feature of viruses is that there exists a positive feedback between the number of replication templates and the number of virally encoded proteins, since viral genomes code for replication-promoting proteins [65]. Consequently, there should be a disproportionate increase in short-term replication efficiency as the copy number of the viral founder genomes within a cell increases. Several lines of evidence support this idea, such as the fact that increased MOI leads to a direct increase in viral gene expression levels in herpesviruses [66]. In polioviruses, phosphatidylserine vesicles, which promote the *en bloc* transmission of multiple virions, increase the rate of viral replication [61], and a similar result was described for noroviruses and rotaviruses [59]. Marseillevirus vesicles have also been shown to accelerate the infection cycle [63], but in this case, the authors speculated that the main reason might be a difference in the mechanism of entry, rather than an effect of coinfection per se.

**Figure 2:**
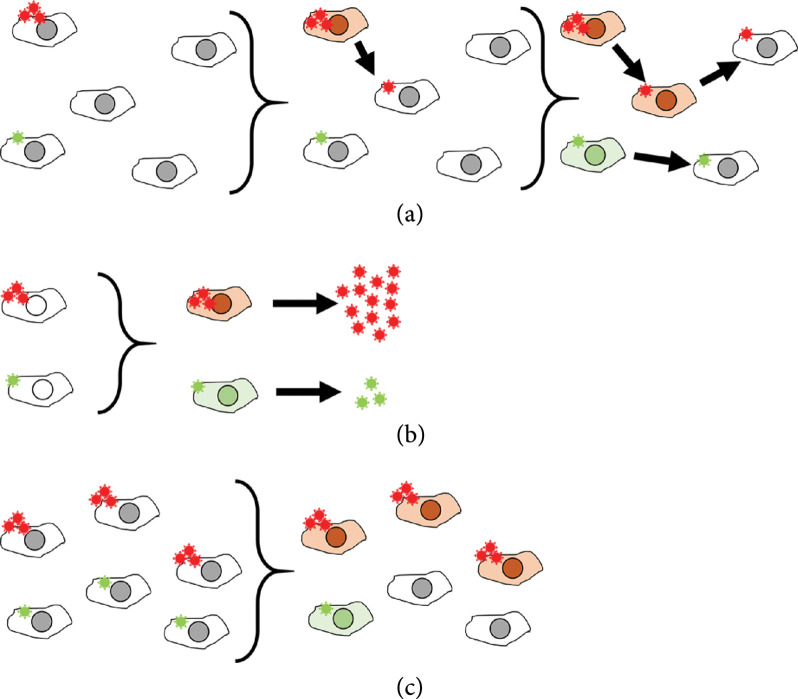
Fitness advantages of coinfection in terms of mass effects. (a) Coinfected cells present a faster replication cycle compared to single infected cell. (b) Coinfected cells produce a higher number in infectious particles (increased per-cell yield). (c) Coinfections are more likely to produce successful infections (increased infectivity).

In addition to simply accelerating the release of viral progeny in infected cells with a high MOI, cooperative replication may increase the viral yield per cell. This could take place if viral progeny production is not limited by cellular resource availability, but by the time an infected cell is productive since viral entry (Figure 2). Apoptosis is triggered in infected cells upon recognition of pathogen-associated molecular patterns such as, for instance, double-stranded RNA [67]. This antiviral response imposes a time window for viruses to produce and release progeny. Increased per-cell yield has been demonstrated for VSV [68], influenza A virus (IAV) [69–72], infectious bursal disease virus (IBDV) [51], and vaccinia virus [73].

Finally, coinfection could also reduce the fraction of abortive infections. When a single virion initiates infection, nucleic acid or protein degradation (spontaneous or driven by cellular mechanisms) may take place before the key steps of viral gene expression or replication can occur. This stochasticity results in abortive infections and contributes to explain why most viral particles fail to yield productive infections (Figure 2). The ratio of productive infections per particle has been shown to increase with the MOI in vaccinia virus [73] and with cell-to-cell transmission in HIV-1 [74], albeit the latter could be due to the use of a different transmission pathway. Polyploid virions of IBDV also show enhanced infectivity [51], but since this is a segmented virus, such increase could be explained by a higher likelihood of transmission of each genome segment.

Despite the fitness benefit that cotransmission mechanisms may provide (Figure 2), the critical question is whether these advantages increase the per-capita yield of viral genomes, compared to single infections. As argued above, K individually infected cells have the potential to yield K times more viral progeny than a single cell coinfected with K particles. Therefore, viruses in coinfected cells should overcome this K-fold cost. To our knowledge, this has only been demonstrated in VSV [68] and vaccinia virus [73]. Coinfection could increase the per-cell viral yield, accelerate the replication cycle [61], or increase infectivity by a factor greater than K. In [59], the authors compared infections with equivalent numbers of free virions and vesicle-cloaked virions, but as they performed *in vivo* infections, it is not easy to determine which cellular-level benefits these vesicles afforded.

There are also connections between the benefits of coinfection and the immune response. IFN is released from infected cells and activates an antiviral state in neighboring cells [75, 76], but the onset of this process takes several hours, at least in cell cultures. Some viruses present specific antiapoptotic proteins that block IFN-activated apoptotic pathways [77, 78], whereas others such as VSV block IFN production [79]. In this context, an increased infection rate or per-cell yield may be critical for infection progression. The fitness advantage of VSV virion aggregates has been shown to correlate with the ability of host cells to mount an effective antiviral innate immune response [68], and similar results have been obtained for IAV [71, 72].

### 2.2. Diversity-Based Interactions

**Figure 3:**
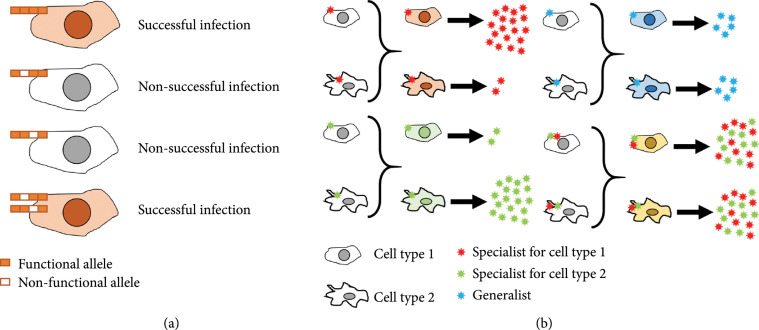
Potential fitness advantages produced by coinfection of cells with different virus variants (diversity-driven interactions). (a) Coinfection can rescue lethal mutants by means of genetic complementation. (b) Genetic complementation can promote the cotransmission of different specialists that are overall fitter than a generalist.

Viruses are the genetic systems exhibiting the highest mutation rates, particularly RNA viruses [80]. This, together with other mechanisms such as recombination, creates highly diverse and rapidly evolving populations at the cost of frequently suffering detrimental or lethal mutations [81]. Coinfection has been speculated to play an important role in determining viral diversity. Genetic complementation among two coinfecting genomes carrying different deleterious mutations may rescue both and allow them to perform successful infections equivalent to a nonmutated virus (Figure 3). However, buffering deleterious effects implies that such mutations will tend to increase their population frequency. Deterministic [82] and stochastic [83] simulations have shown that genetic complementation transiently alleviates the impact of deleterious mutations but does not change mean population fitness over the long term. This is because, at equilibrium, complementation will reduce the average fitness effect of mutations but will similarly increase the frequency of these mutations in the population.

On the other hand, we can speculate that genetic complementation could promote evolvability by increasing cryptic genetic variation. In a context of high MOI and CIU-mediated transmission, genetic complementation might allow deleterious mutations to be maintained in the population and, potentially, some of these mutations could become advantageous in a different context, such as, for instance, in a new host. This could be the case described in [84], where the authors found Asian-type IAV variants in pigs, suggesting that these variants were deleterious but remained at a certain frequency in the original host, potentially due to complementation, and were subsequently amplified in the alternate host where they provided a fitness advantage to the virus.

A potential direct benefit of transmitting genetically diverse viral populations among hosts could take place when viruses must infect different cell types. An often successful strategy in heterogeneous environments is a generalist phenotype that trades off fitness compared to specialists in every specific environment but increases overall performance [85]. We hypothesize that collective viral dispersal could alleviate such trade-offs by allowing different specialist variants to be jointly transmitted (Figure 3). In baculoviruses, OBs carry multiple infectious particles and are essential for host-to-host transmission, as evidenced by the fact that variants carrying a deletion of the polyhedrin gene are unable to infect new hosts. However, OBs containing a mixture of wild-type and polyhedrin-defective variants are efficiently transmitted and can generate more productive and severe infections than genetically homogeneous OBs [86]. This observation suggests that polyhedrin-defective mutants are better adapted to intrahost dissemination, whereas nonmutated genomes provide interhost transmissibility. Both variants could benefit from this interaction, since the enhanced severity of the infection caused by the polyhedrin-defective mutant could potentially favor transmission. Thus, collective viral transmission via OBs could promote a stable coexistence between complementary specialists. The conditions required for such coexistence include functional complementary, synergistic advantages, and a positive assortment of specialists [87]. Further experiments are required to analyze whether this type of interactions in baculoviruses and potentially other viruses exhibits negative frequency-dependent selection, which is another process promoting stable coexistence.

In a similar vein, it has recently been speculated that high-MOI regimes might be at the origin of segmentation in viruses [88], as each segment functions as a defective genome incapable of performing a successful infection alone. For a set of genomes that are constantly cotransmitted, the selection pressure to keep the entire genome as a unit should be reduced, which may lead to some of them retaining only a subset of all functional genes. Eventually, these genomes might experience long deletions and become segments of the original genome. This was experimentally addressed by passaging foot-and-mouth disease virus (FMDV) at high MOIs for many viral generations, which resulted in the emergence of different mutants that could reciprocally complement their genetic defects [89].

## 3. Interactions Mediated by Sequential Infections

Superinfection occurs when a given host or cell is sequentially infected with the same virus or different viruses. As mentioned in Introduction, many viruses possess mechanism to avoid superinfection [13–17]. Intuitively, SIE can be thought of as a trait that helps viruses avoid competition for cellular resources from other incoming viruses. However, it has also been suggested that SIE may function as a cooperative trait. In vaccinia virus, repulsion of virions from the surface of already infected cells was shown to accelerate the propagation of the infection by helping the excluded virions to reach uninfected susceptible cells in the neighborhood [90]. In other cases, SIE may also simply be a passive process, that is, a by-product of infection progression, as the case of Sonchus yellow web nucleorhabdovirus indicates [91].

A seemingly cooperative interaction mediated by sequential infection was demonstrated in bacteriophages. Some phages inhibit the bacterial CRISPR system using anti-CRISPR proteins (Acr), but Acr function is often not sufficient to ensure successful infection, as the CRISPR system is not fully blocked. Still, infection with Acr-encoding phages can induce a transient “immunosuppressed” state in the bacterial cell, in which CRISPR function is partially disabled. As a result, a second Acr phage infecting the same cell may encounter more permissive conditions for infection [92, 93]. This might not be considered superinfection, since primary infections are unsuccessful. In fact, it has been suggested that the phages performing the first abortive infection function as altruistic cooperators.

## 4. Interactions Established between Viruses Infecting Different Cells

Recent work has revealed virus-virus interactions mediated by signals released from infected cells, which are sensed by other infected cells and influence infection outcome. The VSV matrix protein (M protein) inhibits overall host gene expression by binding to nuclear pores and blocking the export of mRNA from the nucleus to the cytoplasm [94]. Such blockade is potentially costly for the virus, for instance, by inducing premature apoptosis, but has the obvious benefit of preventing an antiviral innate immune response. However, this benefit is perceived not only by viruses in the infected cell (autocrine effects) but also by viruses infecting neighbor cells (paracrine effects). Thus, IFN blockade can be considered as a social trait, since it modifies the fitness of other members of the viral population. Importantly, a mutant virus that does not block IFN production can take advantage from the blockade exerted by the normal virus without paying the associated costs. Conversely, the IFN-blocking virus could be suppressed by the presence of an IFN-inducing mutant in the neighborhood. Such costly cooperation has been modeled in the classical evolutionary literature using Hamilton’s rule, which states that the cooperative trait will be favored by selection depending on the direct costs/benefits experienced by the actor, the indirect benefits experienced by other members of the population, and the genetic relatedness between the interacting individuals (Box 1) [95–97]. It was shown that IFN blockade in VSV obeys these principles and, specifically, that the fitness benefit of this trait correlates with genetic relatedness which, in viruses, depends essentially on the spatial structure of the population [24]. In structured infections where VSV virus spread took place in foci, the wild-type IFN blocking virus was vastly superior to a mutant defective for this trait, whereas this advantage was lost in unstructured infections in which the two virus variants were spatially mixed.

**Box 1: Social games between two actors.**
2×2 general payoff matrix for interactions between two phenotypes: C: cooperators and D: cheaters. Detailed analysis of these models can be found in [95–97, 108, 112, 113].

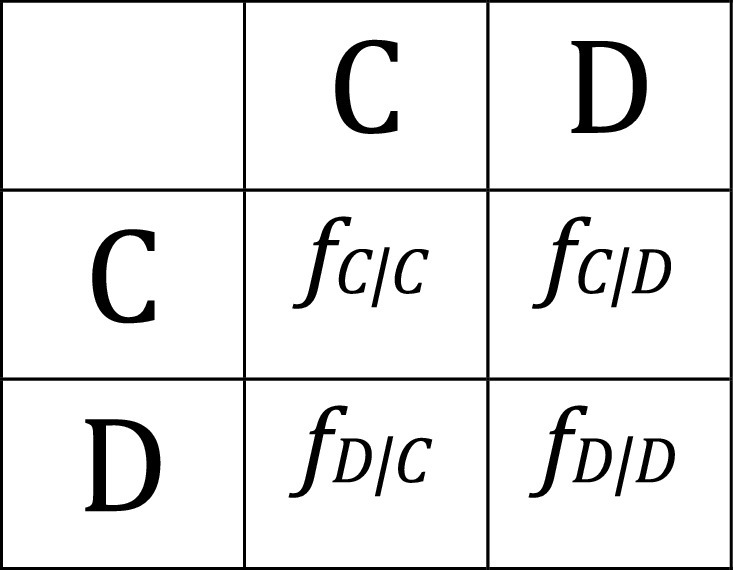

Fitness of cooperators interacting with cooperators: $f_{C\left|C\right.}$
Fitness of cooperators interacting with cheaters: $f_{C\left|D\right.}$
Fitness of cheaters interacting with cooperators: $f_{D\left|C\right.}$
Fitness of cheaters interacting with cheaters: $f_{D\left|D\right.}$
Fraction of cooperators: $p=C/\left(C+D\right)$
Total fitness of cooperators: $f_{C}=f_{C\left|C\right.}p+f_{C\left|D\right.}\left(1-p\right)$
Total fitness of cheaters:$\;f_{D}=f_{D\left|C\right.}p+f_{D\left|D\right.}\left(1-p\right)$
Mean population fitness after cooperation:$\;\bar{f}=f_{C}p+f_{D}\left(1-p\right)$

**Figure 4:**
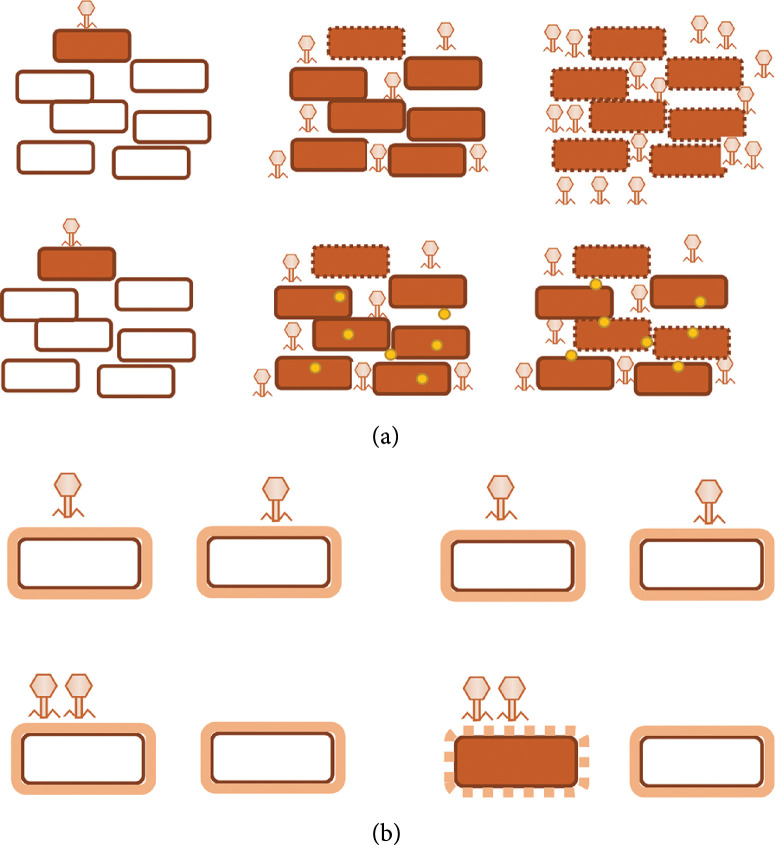
Social interaction of phages. (a) Induced lysogeny increases the fitness of the population, avoiding extinction of the host bacteria. (b) Cooperative depolymerization of bacterial exopolysaccharides allow infections.

Intercellular virus-virus interactions have also been demonstrated in bacteriophages. In recent years, a regulatory system similar to quorum sensing, called Arbitrium, has been described in some temperate *Bacillus* phages such as phi3T or SPBeta. Arbitrium controls lysis versus lysogeny decisions, as well lysogen reactivation [98]. The system comprises at least three genes: a signal peptide (*aimP*), a receptor for that peptide that functions as a transcription factor (*aimR*), and a regulator (*aimX*) that inhibits the expression of prolysogenic genes. When the fraction of infected bacterial hosts is low, there is little signal peptide in the medium, since the peptide is produced by lysogens. In this scenario, *aimR* promotes the expression of *aimX*, which blocks lysogeny genes. This leads to an acute infection phase, with abundant lysis, therefore promoting the accumulation of infectious particles in the medium and, consequently, increasing the fraction of infected hosts. As the density of lysogens increases, so does the concentration of the signal peptide in the medium, which blocks *aimR*, promoting lysogeny. In this way, phages display a communication system to regulate virulence and promote prudent exploitation of the host (Figure 4). However, it is possible for more rapacious variants to emerge and outcompete prudent phages by infecting and lysing as many hosts as possible, leading to the dilemma of parasite prudence, which is actually a particular case of a more general dilemma often referred to as *yield* versus *rate*. Exploiting resources quickly confers a short-term advantage and prevents others from using them but usually reduces efficiency over the long term by exhausting resources [99, 100]. Further investigation is required to analyze what kind of social games is promoted by the Arbitrium system.

Depolymerases may also mediate intercellular virus-virus interactions in phages. These enzymes are typically located on bacteriophage tails and promote infection by disrupting bacterial exopolysaccharides and exposing phage receptors [101–105]. Diffusible depolymerases that are dissociated from tails or remain associated to broken tails could function as classical public goods since they can potentially benefit many members of the local phage population, regardless the specific phage that produced them [104]. It has been suggested that certain synergistic interactions between different phage species at the level of host entry might be mediated by depolymerases [105], although the experimental support for this type of interaction is scarce (Figure 4).

## 5. Viral Cheating

Just as cooperation is widespread in nature, so are cheaters, defined as individuals that reap the benefits of cooperation without contributing to such benefits. If cooperation entails a cost (for instance, producing a capsid), cheaters may experience higher fitness than cooperators because they do not pay such a cost, potentially jeopardizing the maintenance of cooperative traits in the population (the so-called tragedy of the commons). Box 2 illustrates some well-studied cooperative interactions from a game theory point of view. A classical scenario is the Prisoner’s dilemma (PD), in which (i) cooperative traits bear a cost for the actor (–c) and a greater benefit for the receptor (b>c); (ii) the payoff of interactions between cooperators is the sum of both terms $\left(f_{C\left|C\right.}=b–c\right)$; (iii) the payoff of cheaters interacting with cooperators is simply the positive effect they receive from cooperators $\left(f_{D\left|C\right.}=b\right);$ (iv) the payoff of cooperators interacting with cheaters equals the cost of cooperating $\left(f_{C\left|D\right.}=–c\right)$; and (v) the payoff of cheaters interacting with cheaters is zero since there is no interaction $\left(f_{D\left|D\right.}=0\right)$. Thus, the fitness of cooperators is lower than that of cheaters regardless of the probability of encountering a cooperator or a cheater $\left(f_{D\left|C\right.}>f_{C\left|C\right.}\right.$ and $\left.f_{D\left|D\right.}>f_{C\left|D\right.}\right)$. The overall fitness difference between cooperators and cheaters is $f_{C}–f_{D}=-c$, which means that cooperation should always go extinct despite the fact that mean population fitness would be maximal if all individuals were cooperators (Box 3).

**Box 2: Prisoner’s dilemma.**
Cooperators pay a fitness cost (c), while cheaters do not. Any individual interacting with a cooperator obtains a fitness benefit (b), with b>c.

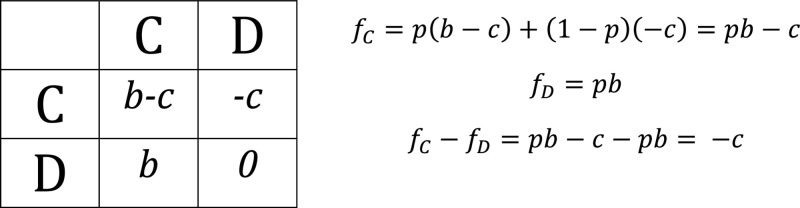

Cooperation is not stable in a PD scenario, although the mean population fitness is maximal in a cooperator-only population: p=1 leads to $\bar{f}=f_{C\left|C\right.}=b-c$.

**Box 3: Snowdrift.**
The cost of cheaters interacting with cheaters (d) is greater than the cost paid by cooperators (d>c).

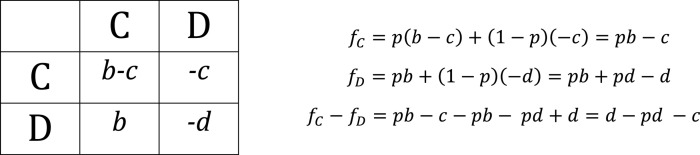

A stable equilibrium point arises at $p=\left(d-c\right)/d$. For cooperation to be stable, the mean population fitness after cooperation must be greater than 0. This is achieved when: $\left(pb-c\right)\times p+\left(pb+pd-d\right)\times \left(1-p\right)>0$, being $p=\left(d-c\right)/d$.

Viruses and genetic systems in general have to accomplish a balance between two key processes, namely, gene expression and replication. During coinfections, a PD social game may occur if the gene products of one virus can be exploited by the other virus [106, 107]. If this is the case, a virus variant that reduces transcription and prioritizes replication may be capable of producing progeny genomes faster than a counterpart that invests more in transcription. However, a cell coinfected with only such cheaters would produce little progeny [81]. PD is fulfilled in this scenario since infections with only cooperators are the fittest, but coinfections between cooperators and cheaters are always dominated by cheaters.

A different scenario arises when the fitness payoffs of the interaction between cheaters (due to an extremely low propensity for transcription) decrease so much that it is even lower than those of single infections (no interaction). Here, a fundamental modification of the payoff matrix occurs, since now, the interaction between cheaters is no longer neutral $\left(f_{D\left|D\right.}=-d<-c\right)$. A new social game called Snowdrift (SD) emerges, in which the optimal strategy is no longer to always be a cheater [108]. Since $f_{D\left|C\right.}>f_{C\left|C\right.}$, but $f_{C\left|D\right.}>f_{D\left|D\right.}$, selection is frequency-dependent and a stable equilibrium between cooperators and cheaters is possible (Box 4). This model describes a very well-known type of cheaters, namely, defective interfering particles (DIPs). DIPs are viruses that have lost a large fraction of the viral genome, such that they are unable to complete an infectious cycle except if a normal or “helper” virus is present in the same cell [109–111]. It has been shown that a nonlinear trade-off between replication and transcription can also generate a SD game between cheater and cooperator viruses [112].

**Box 4: Kin selection.**
A fraction (r) of cooperators interact preferentially with cooperators.

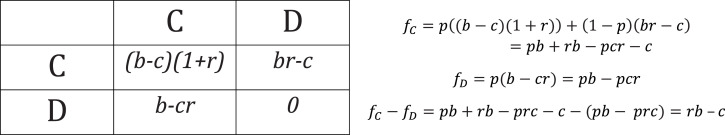

Cooperation may evolve if r>c/b, which is the condition of the well-known Hamilton rule.

Generally speaking, cooperators can outcompete cheaters if they are more likely to interact with other cooperators than cheaters are (Box 1). Hamilton’s rule states that cooperative traits will be favored by selection if r>c/b, where r is the genetic relatedness between interactors for the relevant trait in question, that is, the probability that the benefits of cooperation are received by individuals who share the cooperative trait [95–97]. In the absence of complex phenotype recognition mechanisms such as memory and learning, viruses have to rely on spatial population structure as the main process that promotes genetic relatedness and thus prevents the spread of cheaters.

**Figure 5:**
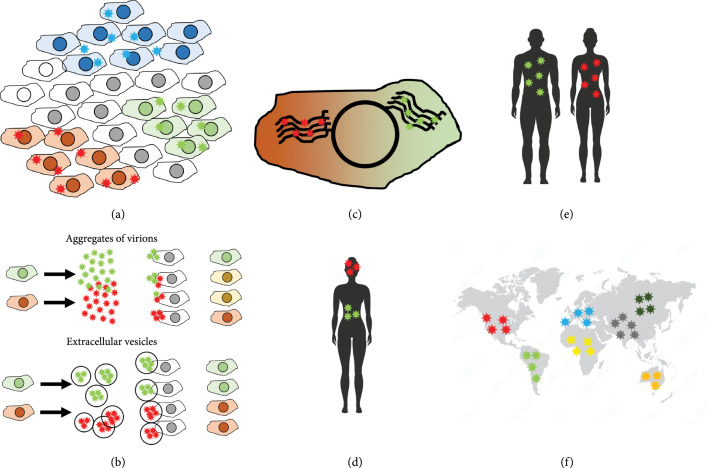
Levels of population structure in viruses. (a) Growth in foci segregates variants due to limited diffusion and superinfection exclusion. (b) CIUs such as extracellular vesicles cause segregation, in contrast to other types of CIUs such as aggregates of virions. (c) Subcellular replication centers. (d) Tissue and organ segregation. (e) Host segregation. (f) Host population segregation.

Viruses exhibit spatial population structure at different levels, ranging from subcellular replication centers to tissues, organs, hosts, or host populations (Figure 5). For instance, infections in solid tissues usually progresses as foci founded by one or a few particles. This creates a region in which each cell is infected by genetically related viruses and where coinfection is likely due to the high local MOI (Figure 5). Concerning CIUs, some allow greater levels of genetic relatedness than others. For instance, extracellular vesicles or occlusion bodies promote the cotransmission of genomes originating from the same infected cell, limiting interactions to “sibling” genomes. Direct cell-to-cell transmission also preserves genetic relatedness in a similar way. In contrast, other CIUs such as virion aggregates generate mixtures of virions from different cells. Increased genetic relatedness should promote the evolution of cooperation between similar variants, but it may also have a negative effect on diversity-driven cooperation.

## 6. Conclusions

The evolution of cooperation is a widely explored topic in the evolutionary biology literature [113]. In the last two decades, the attention has focused on the social interactions displayed by microorganisms, mainly bacteria but more recently also viruses [114, 115]. Cooperative interactions have been shown to have an important effect on viral evolution, such as, for instance, on the evolution of innate immunity evasion [24], but also on infection outcomes in animal [86] and plant viruses [116, 117]. Moreover, the fact that DIPs are found naturally in human patients [118, 119] provides evidence that cooperative interactions take place in nature and have a practical relevance. Although still poorly explored, social evolution could also offer a new perspective on other important traits such as viral tropism and the regulation of virulence. Concerning collective viral transmission, unresolved questions remain, such as whether there is a general fitness benefit for this transmission mode, which should be associated to elevating the MOI, or whether CIUs have evolved in different viruses in response to specific selective pressures. It is noteworthy that extracellular vesicles, which are a well-studied type of CIU vehicle, are used by different viral families such as *Reoviridae* [59], *Caliciviridae* [59], *Picornaviridae* [60], and *Marseilleviridae* [63]. Further research from an evolutionary approach is required to shed light on these unresolved questions.

## References

[1] N. Jorba, R. Coloma, and J. Ortín, “Genetic trans-complementation establishes a new model for influenza virus RNA transcription and replication,” *PLoS Pathogens*, vol. 5, no. 5, article e1000462, 2009 10.1371/journal.ppat.1000462 PMC2682650 19478885

[2] T. Zhou, H. Zhang, T. Lai, C. Qin, N. Shi, H. Wang, M. Jin, S. Zhong, Z. Fan, Y. Liu, Z. Wu, S. Jackson, J. J. Giovannoni, D. Rolin, P. Gallusci, and Y. Hong, “Virus-induced gene complementation reveals a transcription factor network in modulation of tomato fruit ripening,” *Scientific Reports*, vol. 2, no. 1, p. 836, 2012 23150786 10.1038/srep00836 PMC3495281

[3] J. Závada, “Viral pseudotypes and phenotypic mixing,” *Archives of Virology*, vol. 50, no. 1-2, pp. 1–15, 1976 816332 10.1007/BF01317996

[4] J. L. Certo, B. F. Shook, P. D. Yin, J. T. Snider, and W. S. Hu, “Nonreciprocal pseudotyping: murine leukemia virus proteins cannot efficiently package spleen necrosis virus-based vector RNA,” *Journal of Virology*, vol. 72, no. 7, pp. 5408–5413, 1998 9620995 10.1128/jvi.72.7.5408-5413.1998 PMC110171

[5] S. Funke, I. C. Schneider, S. Glaser, M. D. Mühlebach, T. Moritz, R. Cattaneo, K. Cichutek, and C. J. Buchholz, “Pseudotyping lentiviral vectors with the wild-type measles virus glycoproteins improves titer and selectivity,” *Gene Therapy*, vol. 16, no. 5, pp. 700–705, 2009 19212424 10.1038/gt.2009.11

[6] A. Duvergé, and M. Negroni, “Pseudotyping lentiviral vectors: when the clothes make the virus,” *Viruses*, vol. 12, no. 11, p. 1311, 2020 33207797 10.3390/v12111311 PMC7697029

[7] Q. Wu, L. Fang, X. Wu, B. Li, R. Luo, Z. Yu, M. Jin, H. Chen, and S. Xiao, “A pseudotype baculovirus-mediated vaccine confers protective immunity against lethal challenge with H5N1 avian influenza virus in mice and chickens,” *Molecular Immunology*, vol. 46, no. 11-12, pp. 2210–2217, 2009 19446339 10.1016/j.molimm.2009.04.017

[8] P. D. Friesen, and M. S. Nissen, “Gene organization and transcription of TED, a lepidopteran retrotransposon integrated within the baculovirus genome,” *Molecular and Cellular Biology*, vol. 10, no. 6, pp. 3067–3077, 1990 1692964 10.1128/mcb.10.6.3067-3077.1990 PMC360671

[9] C. Hertig, B. E. Coupar, A. R. Gould, and D. B. Boyle, “Field and vaccine strains of fowlpox virus carry integrated sequences from the avian retrovirus, reticuloendotheliosis virus,” *Virology*, vol. 235, no. 2, pp. 367–376, 1997 9281517 10.1006/viro.1997.8691

[10] J. Wang, J. Meers, P. B. Spradbrow, and W. F. Robinson, “Evaluation of immune effects of fowlpox vaccine strains and field isolates,” *Veterinary Microbiology*, vol. 116, no. 1-3, pp. 106–119, 2006 16650660 10.1016/j.vetmic.2006.03.012

[11] A. D. Yurochko, S. M. Huong, and E. S. Huang, “Identification of human cytomegalovirus target sequences in the human immunodeficiency virus long terminal repeat. Potential role of IE2-86 binding to sequences between -120 and -20 in promoter transactivation,” *Journal of Human Virology*, vol. 2, pp. 81–90, 1999 10225210

[12] A. H. Lee, K. J. Lee, S. Kim, and Y. C. Sung, “Transactivation of human immunodeficiency virus type 1 long terminal repeat-directed gene expression by the human foamy virus bel1 protein requires a specific DNA sequence,” *Journal of Virology*, vol. 66, no. 5, pp. 3236–3240, 1992 1313928 10.1128/jvi.66.5.3236-3240.1992 PMC241094

[13] R. H. Adams, and D. T. Brown, “BHK cells expressing Sindbis virus-induced homologous interference allow the translation of nonstructural genes of superinfecting virus,” *Journal of Virology*, vol. 54, no. 2, pp. 351–357, 1985 3989908 10.1128/jvi.54.2.351-357.1985 PMC254804

[14] M. Nethe, B. Berkhout, and A. C. van der Kuyl, “Retroviral superinfection resistance,” *Retrovirology*, vol. 2, no. 1, p. 52, 2005 16107223 10.1186/1742-4690-2-52 PMC1224871

[15] T. Schaller, N. Appel, G. Koutsoudakis, S. Kallis, V. Lohmann, T. Pietschmann, and R. Bartenschlager, “Analysis of hepatitis C virus superinfection exclusion by using novel fluorochrome gene-tagged viral genomes,” *Journal of Virology*, vol. 81, no. 9, pp. 4591–4603, 2007 17301154 10.1128/JVI.02144-06 PMC1900174

[16] C. Dietrich, and E. Maiss, “Fluorescent labelling reveals spatial separation of potyvirus populations in mixed infected Nicotiana benthamiana plants,” *The Journal of General Virology*, vol. 84, no. 10, pp. 2871–2876, 2003 13679622 10.1099/vir.0.19245-0

[17] J. Bondy-Denomy, J. Qian, E. R. Westra, A. Buckling, D. S. Guttman, A. R. Davidson, and K. L. Maxwell, “Prophages mediate defense against phage infection through diverse mechanisms,” *The ISME Journal*, vol. 10, no. 12, pp. 2854–2866, 2016 27258950 10.1038/ismej.2016.79 PMC5148200

[18] S. Folimonova, “Developing an understanding of cross-protection by Citrus tristeza virus,” *Frontiers in Microbiology*, vol. 4, 2013 10.3389/fmicb.2013.00076 PMC3616238 23577008

[19] X.-F. Zhang, S. Zhang, Q. Guo, R. Sun, T. Wei, and F. Qu, “A new mechanistic model for viral cross protection and superinfection exclusion,” *Frontiers in Plant Science*, vol. 9, 2018 10.3389/fpls.2018.00040 PMC5788904 29422912

[20] T. DaPalma, B. P. Doonan, N. M. Trager, and L. M. Kasman, “A systematic approach to virus-virus interactions,” *Virus Research*, vol. 149, no. 1, pp. 1–9, 2010 20093154 10.1016/j.virusres.2010.01.002 PMC7172858

[21] A. Biancotto, S. J. Iglehart, A. Lisco, C. Vanpouille, J. C. Grivel, N. S. Lurain, P. S. Reichelderfer, and L. B. Margolis, “Upregulation of human cytomegalovirus by HIV type 1 in human lymphoid tissue ex vivo,” *AIDS Research and Human Retroviruses*, vol. 24, no. 3, pp. 453–462, 2008 18327985 10.1089/aid.2007.0155

[22] C. Celum, R. Levine, M. Weaver, and A. Wald, “Genital herpes and human immunodeficiency virus: double trouble,” *Bulletin of the World Health Organization*, vol. 82, no. 6, pp. 447–453, 2004 15356938 PMC2622854

[23] J. S. Sheffield, G. D. Wendel, D. D. McIntire, and M. V. Norgard, “Effect of genital ulcer disease on HIV-1 coreceptor expression in the female genital tract,” *The Journal of Infectious Diseases*, vol. 196, no. 10, pp. 1509–1516, 2007 18008231 10.1086/522518

[24] P. Domingo-Calap, E. Segredo-Otero, M. Durán-Moreno, and R. Sanjuán, “Social evolution of innate immunity evasion in a virus,” *Nature Microbiology*, vol. 4, no. 6, pp. 1006–1013, 2019 10.1038/s41564-019-0379-8 PMC6544518 30833734

[25] K. Dee, D. M. Goldfarb, J. Haney, J. A. R. Amat, V. Herder, M. Stewart, A. M. Szemiel, M. Baguelin, and P. R. Murcia, “Human rhinovirus infection blocks severe acute respiratory syndrome coronavirus 2 replication within the respiratory epithelium: implications for COVID-19 epidemiology,” *The Journal of Infectious Diseases*, vol. 224, no. 1, pp. 31–38, 2021 33754149 10.1093/infdis/jiab147 PMC8083659

[26] S. Nickbakhsh, C. Mair, L. Matthews, R. Reeve, P. C. D. Johnson, F. Thorburn, B. von Wissmann, A. Reynolds, J. McMenamin, R. N. Gunson, and P. R. Murcia, “Virus–virus interactions impact the population dynamics of influenza and the common cold,” *PNAS*, vol. 116, no. 52, pp. 27142–27150, 2019 31843887 10.1073/pnas.1911083116 PMC6936719

[27] L. Chao, K. A. Hanley, C. L. Burch, C. Dahlberg, and P. E. Turner, “Kin selection and parasite evolution: higher and lower virulence with hard and soft selection,” *The Quarterly Review of Biology*, vol. 75, no. 3, 2000 10.1086/393499 11008699

[28] H. Y. Chen, M. Di Mascio, A. S. Perelson, D. D. Ho, and L. Zhang, “Determination of virus burst size in vivo using a single-cycle SIV in rhesus macaques,” *PNAS*, vol. 104, no. 48, pp. 19079–19084, 2007 18025463 10.1073/pnas.0707449104 PMC2141911

[29] S. J. Stray, and G. M. Air, “Apoptosis by influenza viruses correlates with efficiency of viral mRNA synthesis,” *Virus Research*, vol. 77, no. 1, pp. 3–17, 2001 11451482 10.1016/s0168-1702(01)00260-x

[30] I. S. Novella, L. A. Ball, and G. W. Wertz, “Fitness analyses of vesicular stomatitis strains with rearranged genomes reveal replicative disadvantages,” *Journal of Virology*, vol. 78, no. 18, pp. 9837–9841, 2004 15331718 10.1128/JVI.78.18.9837-9841.2004 PMC514966

[31] T. V. Stirbat, A. Mgharbel, S. Bodennec, K. Ferri, H. C. Mertani, J. P. Rieu, and H. Delanoë-Ayari, “Fine tuning of tissues’ viscosity and surface tension through contractility suggests a new role for *α*-catenin,” *PLoS One*, vol. 8, no. 2, article e52554, 2013 10.1371/journal.pone.0052554 PMC3563668 23390488

[32] Z. J. Storms, E. Arsenault, D. Sauvageau, and D. G. Cooper, “Bacteriophage adsorption efficiency and its effect on amplification,” *Bioprocess and Biosystems Engineering*, vol. 33, no. 7, pp. 823–831, 2010 20066440 10.1007/s00449-009-0405-y

[33] Y. Shao, and I.-N. Wang, “Bacteriophage adsorption rate and optimal lysis time,” *Genetics*, vol. 180, no. 1, pp. 471–482, 2008 18757924 10.1534/genetics.108.090100 PMC2535697

[34] S. Kawakami, Y. Watanabe, and R. N. Beachy, “Tobacco mosaic virus infection spreads cell to cell as intact replication complexes,” *PNAS*, vol. 101, no. 16, pp. 6291–6296, 2004 15079061 10.1073/pnas.0401221101 PMC395962

[35] D. Kumar, R. Kumar, T. K. Hyun, and J.-Y. Kim, “Cell-to-cell movement of viruses via plasmodesmata,” *Journal of Plant Research*, vol. 128, no. 1, pp. 37–47, 2015 25527904 10.1007/s10265-014-0683-6

[36] S. Gutiérrez, M. Yvon, G. Thébaud, B. Monsion, Y. Michalakis, and S. Blanc, “Dynamics of the multiplicity of cellular infection in a plant virus,” *PLoS Pathogens*, vol. 6, no. 9, article e1001113, 2010 10.1371/journal.ppat.1001113 PMC2940754 20862320

[37] S. Gutiérrez, E. Pirolles, M. Yvon, V. Baecker, Y. Michalakis, and S. Blanc, “The multiplicity of cellular infection changes depending on the route of cell infection in a plant virus,” *Journal of Virology*, vol. 89, no. 18, pp. 9665–9675, 2015 26178988 10.1128/JVI.00537-15 PMC4542342

[38] S. Cudmore, P. Cossart, G. Griffiths, and M. Way, “Actin-based motility of vaccinia virus,” *Nature*, vol. 378, no. 6557, pp. 636–638, 1995 8524400 10.1038/378636a0

[39] M. J. Lehmann, N. M. Sherer, C. B. Marks, M. Pypaert, and W. Mothes, “Actin- and myosin-driven movement of viruses along filopodia precedes their entry into cells,” *The Journal of Cell Biology*, vol. 170, no. 2, pp. 317–325, 2005 16027225 10.1083/jcb.200503059 PMC2171413

[40] S. Sowinski, C. Jolly, O. Berninghausen, M. A. Purbhoo, A. Chauveau, K. Köhler, S. Oddos, P. Eissmann, F. M. Brodsky, C. Hopkins, B. Önfelt, Q. Sattentau, and D. M. Davis, “Membrane nanotubes physically connect T cells over long distances presenting a novel route for HIV-1 transmission,” *Nature Cell Biology*, vol. 10, no. 2, pp. 211–219, 2008 18193035 10.1038/ncb1682

[41] K. Takeuchi, N. Miyajima, N. Nagata, M. Takeda, and M. Tashiro, “Wild-type measles virus induces large syncytium formation in primary human small airway epithelial cells by a SLAM(CD150)-independent mechanism,” *Virus Research*, vol. 94, no. 1, pp. 11–16, 2003 12837552 10.1016/s0168-1702(03)00117-5

[42] M.-E. Hamelin, Y. Abed, and G. Boivin, “Human metapneumovirus: a new player among respiratory viruses,” *Clinical Infectious Diseases*, vol. 38, no. 7, pp. 983–990, 2004 15034830 10.1086/382536 PMC7107938

[43] N. L. Cole, and C. Grose, “Membrane fusion mediated by herpesvirus glycoproteins: the paradigm of varicella-zoster virus,” *Reviews in Medical Virology*, vol. 13, no. 4, pp. 207–222, 2003 12820183 10.1002/rmv.377

[44] R. Nardacci, A. Antinori, L. M. Larocca, V. Arena, A. Amendola, J. L. Perfettini, G. Kroemer, and M. Piacentini, “Characterization of cell death pathways in human immunodeficiency virus- associated encephalitis,” *The American Journal of Pathology*, vol. 167, no. 3, pp. 695–704, 2005 16127150 10.1016/S0002-9440(10)62044-5 PMC1698734

[45] R. A. Alvarez, M. I. Barría, and B. K. Chen, “Unique features of HIV-1 spread through T cell virological synapses,” *PLoS Pathogens*, vol. 10, no. 12, article e1004513, 2014 10.1371/journal.ppat.1004513 PMC4270788 25522148

[46] M. Dupont, and Q. J. Sattentau, “Macrophage cell-cell interactions promoting HIV-1 infection,” *Viruses*, vol. 12, no. 5, p. 492, 2020 32354203 10.3390/v12050492 PMC7290394

[47] R. Sanjuán, “Collective infectious units in viruses,” *Trends in Microbiology*, vol. 25, no. 5, pp. 402–412, 2017 28262512 10.1016/j.tim.2017.02.003 PMC5837019

[48] M. J. Iglesias-Sanchez, and C. Lopez-Galindez, “Each genomic RNA in HIV-1 heterozygous virus generate new virions,” *Virology*, vol. 333, no. 2, pp. 316–323, 2005 15801124 10.1016/j.virol.2004.12.027

[49] M. Rager, S. Vongpunsawad, W. P. Duprex, and R. Cattaneo, “Polyploid measles virus with hexameric genome length,” *The EMBO Journal*, vol. 21, no. 10, pp. 2364–2372, 2002 12006489 10.1093/emboj/21.10.2364 PMC126007

[50] D. R. Beniac, P. L. Melito, S. L. deVarennes, S. L. Hiebert, M. J. Rabb, L. L. Lamboo, S. M. Jones, and T. F. Booth, “The organisation of Ebola virus reveals a capacity for extensive, modular polyploidy,” *PLoS One*, vol. 7, no. 1, article e29608, 2012 10.1371/journal.pone.0029608 PMC3256159 22247782

[51] D. Luque, G. Rivas, C. Alfonso, J. L. Carrascosa, J. F. Rodríguez, and J. R. Castón, “Infectious bursal disease virus is an icosahedral polyploid dsRNA virus,” *PNAS*, vol. 106, no. 7, pp. 2148–2152, 2009 19164552 10.1073/pnas.0808498106 PMC2650107

[52] M. Lago, J. F. Rodríguez, I. Bandín, and C. P. Dopazo, “Aquabirnavirus polyploidy: a new strategy to modulate virulence?,” *The Journal of General Virology*, vol. 97, no. 5, pp. 1168–1177, 2016 26902908 10.1099/jgv.0.000434

[53] J. M. Cuevas, M. Durán-Moreno, and R. Sanjuán, “Multi-virion infectious units arise from free viral particles in an enveloped virus,” *Nature Microbiology*, vol. 2, no. 7, p. 17078, 2017 10.1038/nmicrobiol.2017.78 PMC5447809 28530650

[54] V. Anschau, and R. Sanjuán, “Fibrinogen gamma chain promotes aggregation of vesicular stomatitis virus in saliva,” *Viruses*, vol. 12, no. 3, p. 282, 2020 32143369 10.3390/v12030282 PMC7150986

[55] J. Münch, E. Rücker, L. Ständker, K. Adermann, C. Goffinet, M. Schindler, S. Wildum, R. Chinnadurai, D. Rajan, A. Specht, G. Giménez-Gallego, P. C. Sánchez, D. M. Fowler, A. Koulov, J. W. Kelly, W. Mothes, J. C. Grivel, L. Margolis, O. T. Keppler, W. G. Forssmann, and F. Kirchhoff, “Semen-derived amyloid fibrils drastically enhance HIV infection,” *Cell*, vol. 131, no. 6, pp. 1059–1071, 2007 18083097 10.1016/j.cell.2007.10.014

[56] K.-A. Kim, M. Yolamanova, O. Zirafi, N. R. Roan, L. Staendker, W. G. Forssmann, A. Burgener, N. Dejucq-Rainsford, B. H. Hahn, G. M. Shaw, W. C. Greene, F. Kirchhoff, and J. Münch, “Semen-mediated enhancement of HIV infection is donor-dependent and correlates with the levels of SEVI,” *Retrovirology*, vol. 7, no. 1, p. 55, 2010 20573198 10.1186/1742-4690-7-55 PMC2914040

[57] S. Yang, L. Zhao, R. Ma, W. Fang, J. Hu, C. Lei, and X. Sun, “Improving baculovirus infectivity by efficiently embedding enhancing factors into occlusion bodies,” *Applied and Environmental Microbiology*, vol. 83, no. 14, article e00595, 2017 10.1128/AEM.00595-17 PMC5494622 28500037

[58] D. B. Sajjan, and S. B. Hinchigeri, “Structural organization of baculovirus occlusion bodies and protective role of multilayered polyhedron envelope protein,” *Food Environ Virol*, vol. 8, no. 1, pp. 86–100, 2016 26787118 10.1007/s12560-016-9227-7

[59] M. Santiana, S. Ghosh, B. A. Ho, V. Rajasekaran, W. L. du, Y. Mutsafi, D. A. de Jésus-Diaz, S. V. Sosnovtsev, E. A. Levenson, G. I. Parra, P. M. Takvorian, A. Cali, C. Bleck, A. N. Vlasova, L. J. Saif, J. T. Patton, P. Lopalco, A. Corcelli, K. Y. Green, and N. Altan-Bonnet, “Vesicle-cloaked virus clusters are optimal units for inter-organismal viral transmission,” *Cell Host & Microbe*, vol. 24, no. 2, pp. 208–220.e8, 2018 30092198 10.1016/j.chom.2018.07.006 PMC6226266

[60] J.-V. Bou, R. Geller, and R. Sanjuán, “Membrane-associated enteroviruses undergo intercellular transmission as pools of sibling viral genomes,” *Cell Reports*, vol. 29, no. 3, pp. 714–723.e4, 2019 31618638 10.1016/j.celrep.2019.09.014 PMC6899498

[61] Y.-H. Chen, W. L. du, M. C. Hagemeijer, P. M. Takvorian, C. Pau, A. Cali, C. A. Brantner, E. S. Stempinski, P. S. Connelly, H. C. Ma, P. Jiang, E. Wimmer, G. Altan-Bonnet, and N. Altan-Bonnet, “Phosphatidylserine vesicles enable efficient en bloc transmission of enteroviruses,” *Cell*, vol. 160, no. 4, pp. 619–630, 2015 25679758 10.1016/j.cell.2015.01.032 PMC6704014

[62] V. Ramakrishnaiah, and L. J. W. van der Laan, “Hepatitis virus hijacks shuttle: exosome-like vesicles provide protection against neutralizing antibodies,” *Hepatology*, vol. 59, no. 6, pp. 2416–2418, 2014 24273053 10.1002/hep.26943

[63] T. S. Arantes, R. A. L. Rodrigues, L. K. dos Santos Silva, G. P. Oliveira, H. L. de Souza, J. Y. B. Khalil, D. B. de Oliveira, A. A. Torres, L. L. da Silva, P. Colson, E. G. Kroon, F. G. da Fonseca, C. A. Bonjardim, B. la Scola, and J. S. Abrahão, “The large Marseillevirus explores different entry pathways by forming giant infectious vesicles,” *Journal of Virology*, vol. 90, no. 11, pp. 5246–5255, 2016 26984730 10.1128/JVI.00177-16 PMC4934737

[64] R. Sanjuán, and M.-I. Thoulouze, “Why viruses sometimes disperse in groups?†,” *Virus Evol*, vol. 5, no. 1, p. vez014, 2019 31249695 10.1093/ve/vez014 PMC6589326

[65] I. Andreu-Moreno, J.-V. Bou, and R. Sanjuán, “Cooperative nature of viral replication,” *Science Advances*, vol. 6, no. 49, p. eabd4942, 2020 33277258 10.1126/sciadv.abd4942 PMC7821885

[66] E. M. Cohen, and O. Kobiler, “Gene expression correlates with the number of herpes viral genomes initiating infection in single cells,” *PLoS Pathogens*, vol. 12, no. 12, article e1006082, 2016 10.1371/journal.ppat.1006082 PMC5161387 27923068

[67] H. Kumar, T. Kawai, and S. Akira, “Pathogen recognition by the innate immune system,” *International Reviews of Immunology*, vol. 30, no. 1, pp. 16–34, 2011 21235323 10.3109/08830185.2010.529976

[68] I. Andreu-Moreno, and R. Sanjuán, “Collective infection of cells by viral aggregates promotes early viral proliferation and reveals a cellular-level Allee effect,” *Current Biology*, vol. 28, no. 20, pp. 3212–3219.e4, 2018 30318351 10.1016/j.cub.2018.08.028 PMC6783297

[69] F. S. Heldt, S. Y. Kupke, S. Dorl, U. Reichl, and T. Frensing, “Single-cell analysis and stochastic modelling unveil large cell-to-cell variability in influenza A virus infection,” *Nature Communications*, vol. 6, no. 1, p. 8938, 2015 10.1038/ncomms9938 PMC4673863 26586423

[70] N. T. Jacobs, N. O. Onuoha, A. Antia, J. Steel, R. Antia, and A. C. Lowen, “Incomplete influenza A virus genomes occur frequently but are readily complemented during localized viral spread,” *Nature Communications*, vol. 10, no. 1, p. 3526, 2019 10.1038/s41467-019-11428-x PMC6684657 31387995

[71] B. E. Martin, J. D. Harris, J. Sun, K. Koelle, and C. B. Brooke, “Cellular co-infection can modulate the efficiency of influenza A virus production and shape the interferon response,” *PLoS Pathogens*, vol. 16, no. 10, article e1008974, 2020 10.1371/journal.ppat.1008974 PMC7592918 33064776

[72] K. L. Phipps, K. Ganti, N. T. Jacobs, C. Y. Lee, S. Carnaccini, M. C. White, M. Manandhar, B. E. Pickett, G. S. Tan, L. M. Ferreri, D. R. Perez, and A. C. Lowen, “Collective interactions augment influenza A virus replication in a host- dependent manner,” *Nature Microbiology*, vol. 5, no. 9, pp. 1158–1169, 2020 10.1038/s41564-020-0749-2 PMC7484227 32632248

[73] P. Stiefel, F. I. Schmidt, P. Dörig, P. Behr, T. Zambelli, J. A. Vorholt, and J. Mercer, “Cooperative vaccinia infection demonstrated at the single-cell level using FluidFM,” *Nano Letters*, vol. 12, no. 8, pp. 4219–4227, 2012 22731659 10.1021/nl3018109

[74] L. M. Agosto, P. D. Uchil, and W. Mothes, “HIV cell-to-cell transmission: effects on pathogenesis and antiretroviral therapy,” *Trends in Microbiology*, vol. 23, no. 5, pp. 289–295, 2015 25766144 10.1016/j.tim.2015.02.003 PMC4417442

[75] M. G. Katze, Y. He, and M. Gale, “Viruses and interferon: a fight for supremacy,” *Nature Reviews. Immunology*, vol. 2, no. 9, pp. 675–687, 2002 10.1038/nri888 12209136

[76] C. E. Samuel, “Antiviral actions of interferons,” *Clinical microbiology reviews*, vol. 14, pp. 778–809, 2001 11585785 10.1128/CMR.14.4.778-809.2001 PMC89003

[77] M. H. Orzalli, and J. C. Kagan, “Apoptosis and necroptosis as host defense strategies to prevent viral infection,” *Trends in Cell Biology*, vol. 27, no. 11, pp. 800–809, 2017 28642032 10.1016/j.tcb.2017.05.007 PMC5653411

[78] F. Nainu, A. Shiratsuchi, and Y. Nakanishi, “Induction of apoptosis and subsequent phagocytosis of virus-infected cells as an antiviral mechanism,” *Frontiers in Immunology*, vol. 8, p. 1220, 2017 29033939 10.3389/fimmu.2017.01220 PMC5624992

[79] E. A. Voigt, A. Swick, and J. Yin, “Rapid induction and persistence of paracrine-induced cellular antiviral states arrest viral infection spread in A549 cells,” *Virology*, vol. 496, pp. 59–66, 2016 27254596 10.1016/j.virol.2016.05.019 PMC5159750

[80] R. Sanjuán, and P. Domingo-Calap, “Mechanisms of viral mutation,” *Cellular and Molecular Life Sciences*, vol. 73, no. 23, pp. 4433–4448, 2016 27392606 10.1007/s00018-016-2299-6 PMC5075021

[81] R. Sanjuán, “Mutational fitness effects in RNA and single-stranded DNA viruses: common patterns revealed by site-directed mutagenesis studies,” *Philosophical Transactions of the Royal Society of London. Series B, Biological Sciences*, vol. 365, no. 1548, pp. 1975–1982, 2010 20478892 10.1098/rstb.2010.0063 PMC2880115

[82] H. Gao, and M. W. Feldman, “Complementation and epistasis in viral coinfection dynamics,” *Genetics*, vol. 182, no. 1, pp. 251–263, 2009 19270273 10.1534/genetics.108.099796 PMC2674821

[83] E. Segredo-Otero, and R. Sanjuán, “The effect of genetic complementation on the fitness and diversity of viruses spreading as collective infectious units,” *Virus Research*, vol. 267, pp. 41–48, 2019 31077765 10.1016/j.virusres.2019.05.005

[84] P. R. Murcia, J. Hughes, P. Battista, L. Lloyd, G. J. Baillie, R. H. Ramirez-Gonzalez, D. Ormond, K. Oliver, D. Elton, J. A. Mumford, M. Caccamo, P. Kellam, B. T. Grenfell, E. C. Holmes, and J. L. N. Wood, “Evolution of an Eurasian avian-like influenza virus in naïve and vaccinated pigs,” *PLoS Pathogens*, vol. 8, no. 5, article e1002730, 2012 10.1371/journal.ppat.1002730 PMC3364949 22693449

[85] V. J. Morley, M. Sistrom, J. A. Usme-Ciro, S. K. Remold, and P. E. Turner, “Evolution in spatially mixed host environments increases divergence for evolved fitness and intrapopulation genetic diversity in RNA viruses,” *Virus Evol*, vol. 2, no. 1, p. vev022, 2016 27774292 10.1093/ve/vev022 PMC4989875

[86] O. Simón, T. Williams, P. Caballero, and M. López-Ferber, “Dynamics of deletion genotypes in an experimental insect virus population,” *Proceedings of the Biological Sciences*, vol. 273, no. 1588, pp. 783–790, 2006 10.1098/rspb.2005.3394 PMC1560231 16618670

[87] S. Giri, S. Waschina, C. Kaleta, and C. Kost, “Defining division of labor in microbial communities,” *Journal of Molecular Biology*, vol. 431, no. 23, pp. 4712–4731, 2019 31260694 10.1016/j.jmb.2019.06.023

[88] S. F. Elena, “Evolutionary transitions during RNA virus experimental evolution,” *Philosophical Transactions of the Royal Society of London. Series B, Biological Sciences*, vol. 371, no. 1701, p. 20150441, 2016 27431519 10.1098/rstb.2015.0441 PMC4958935

[89] J. García-Arriaza, S. C. Manrubia, M. Toja, E. Domingo, and C. Escarmís, “Evolutionary transition toward defective RNAs that are infectious by complementation,” *Journal of Virology*, vol. 78, no. 21, pp. 11678–11685, 2004 15479809 10.1128/JVI.78.21.11678-11685.2004 PMC523252

[90] V. Doceul, M. Hollinshead, L. van der Linden, and G. L. Smith, “Repulsion of superinfecting virions: a mechanism for rapid virus spread,” *Science*, vol. 327, no. 5967, pp. 873–876, 2010 20093437 10.1126/science.1183173 PMC4202693

[91] X. Zhou, K. Sun, X. Zhou, A. O. Jackson, and Z. Li, “The matrix protein of a plant rhabdovirus mediates superinfection exclusion by inhibiting viral transcription,” *Journal of Virology*, vol. 93, no. 20, article e00680, 2019 10.1128/JVI.00680-19 PMC6798102 31341043

[92] M. Landsberger, S. Gandon, S. Meaden, C. Rollie, A. Chevallereau, H. Chabas, A. Buckling, E. R. Westra, and S. van Houte, “Anti-CRISPR phages cooperate to overcome CRISPR-Cas immunity,” *Cell*, vol. 174, no. 4, pp. 908–916.e12, 2018 30033365 10.1016/j.cell.2018.05.058 PMC6086933

[93] A. L. Borges, J. Y. Zhang, M. C. F. Rollins, B. A. Osuna, B. Wiedenheft, and J. Bondy-Denomy, “Bacteriophage cooperation suppresses CRISPR-Cas3 and Cas9 immunity,” *Cell*, vol. 174, no. 4, pp. 917–925.e10, 2018 30033364 10.1016/j.cell.2018.06.013 PMC6086726

[94] J. M. Petersen, L. S. Her, V. Varvel, E. Lund, and J. E. Dahlberg, “The matrix protein of vesicular stomatitis virus inhibits nucleocytoplasmic transport when it is in the nucleus and associated with nuclear pore complexes,” *Molecular and Cellular Biology*, vol. 20, no. 22, pp. 8590–8601, 2000 11046154 10.1128/mcb.20.22.8590-8601.2000 PMC102164

[95] J. Smith, J. D. Van Dyken, and P. Zee, “A generalization of Hamilton’s rule for the evolution of microbial cooperation,” *Science*, vol. 328, no. 5986, pp. 1700–1703, 2010 20576891 10.1126/science.1189675 PMC3097903

[96] A. F. G. Bourke, “Hamilton’s rule and the causes of social evolution,” *Philosophical Transactions of the Royal Society of London. Series B, Biological Sciences*, vol. 369, no. 1642, p. 20130362, 2014 24686934 10.1098/rstb.2013.0362 PMC3982664

[97] S. A. West, A. S. Griffin, and A. Gardner, “Evolutionary explanations for cooperation,” *Current Biology*, vol. 17, no. 16, pp. R661–R672, 2007 17714660 10.1016/j.cub.2007.06.004

[98] Z. Erez, I. Steinberger-Levy, M. Shamir, S. Doron, A. Stokar-Avihail, Y. Peleg, S. Melamed, A. Leavitt, A. Savidor, S. Albeck, G. Amitai, and R. Sorek, “Communication between viruses guides lysis-lysogeny decisions,” *Nature*, vol. 541, no. 7638, pp. 488–493, 2017 28099413 10.1038/nature21049 PMC5378303

[99] M. Boots, and M. Mealor, “Local interactions select for lower pathogen infectivity,” *Science*, vol. 315, no. 5816, pp. 1284–1286, 2007 17332415 10.1126/science.1137126

[100] A. Buckling, and M. A. Brockhurst, “Kin selection and the evolution of virulence,” *Heredity*, vol. 100, no. 5, pp. 484–488, 2008 18212805 10.1038/sj.hdy.6801093

[101] A. C. Hernandez-Morales, L. L. Lessor, T. L. Wood, D. Migl, E. M. Mijalis, J. Cahill, W. K. Russell, R. F. Young, and J. J. Gill, “Genomic and biochemical characterization of Acinetobacter podophage petty reveals a novel lysis mechanism and tail-associated depolymerase activity,” *Journal of Virology*, vol. 92, no. 6, article e01064, 2018 10.1128/JVI.01064-17 PMC5827379 29298884

[102] L. Mi, Y. Liu, C. Wang, T. He, S. Gao, S. Xing, Y. Huang, H. Fan, X. Zhang, W. Yu, Z. Mi, Y. Tong, C. Bai, and F. Han, “Identification of a lytic Pseudomonas aeruginosa phage depolymerase and its anti-biofilm effect and bactericidal contribution to serum,” *Virus Genes*, vol. 55, no. 3, pp. 394–405, 2019 30937696 10.1007/s11262-019-01660-4

[103] Z. Guo, J. Huang, G. Yan, L. Lei, S. Wang, L. Yu, L. Zhou, A. Gao, X. Feng, W. Han, J. Gu, and J. Yang, “Identification and characterization of Dpo42, a novel depolymerase derived from the Escherichia coli phage vB_EcoM_ECOO78,” *Frontiers in Microbiology*, vol. 8, p. 1460, 2017 28824588 10.3389/fmicb.2017.01460 PMC5539073

[104] A. Latka, B. Maciejewska, G. Majkowska-Skrobek, Y. Briers, and Z. Drulis-Kawa, “Bacteriophage-encoded virion-associated enzymes to overcome the carbohydrate barriers during the infection process,” *Applied Microbiology and Biotechnology*, vol. 101, no. 8, pp. 3103–3119, 2017 28337580 10.1007/s00253-017-8224-6 PMC5380687

[105] M. Schmerer, I. J. Molineux, and J. J. Bull, “Synergy as a rationale for phage therapy using phage cocktails,” *PeerJ*, vol. 2, article e590, 2014 10.7717/peerj.590 PMC4179555 25279269

[106] H. C. Gelderblom, D. N. Vatakis, S. A. Burke, S. D. Lawrie, G. C. Bristol, and D. N. Levy, “Viral complementation allows HIV-1 replication without integration,” *Retrovirology*, vol. 5, no. 1, p. 60, 2008 18613957 10.1186/1742-4690-5-60 PMC2474848

[107] N. Appel, U. Herian, and R. Bartenschlager, “Efficient rescue of hepatitis C virus RNA replication by trans-complementation with nonstructural protein 5A,” *Journal of Virology*, vol. 79, no. 2, pp. 896–909, 2005 15613318 10.1128/JVI.79.2.896-909.2005 PMC538567

[108] M. Doebeli, and C. Hauert, “Models of cooperation based on the Prisoner’s Dilemma and the snowdrift game: Prisoner’s Dilemma and the Snowdrift game,” *Ecology letters*, vol. 8, pp. 748–766, 2005

[109] E. Szathmáry, “Natural selection and dynamical coexistence of defective and complementing virus segments,” *Journal of Theoretical Biology*, vol. 157, no. 3, pp. 383–406, 1992 1465021 10.1016/s0022-5193(05)80617-4

[110] T. B. Kirkwood, and C. R. Bangham, “Cycles, chaos, and evolution in virus cultures: a model of defective interfering particles,” *PNAS*, vol. 91, no. 18, pp. 8685–8689, 1994 8078942 10.1073/pnas.91.18.8685 PMC44671

[111] C. R. Bangham, and T. B. Kirkwood, “Defective interfering particles: effects in modulating virus growth and persistence,” *Virology*, vol. 179, no. 2, pp. 821–826, 1990 2238471 10.1016/0042-6822(90)90150-p

[112] L. Chao, and S. F. Elena, “Nonlinear trade-offs allow the cooperation game to evolve from Prisoner’s Dilemma to Snowdrift,” *Proceedings of the Royal Society B: Biological Sciences*, vol. 284, no. 1854, p. 20170228, 2017 10.1098/rspb.2017.0228 PMC5443946 28490625

[113] S. A. Frank, “Natural selection. VII. History and interpretation of kin selection theory,” *Journal of Evolutionary Biology*, vol. 26, no. 6, pp. 1151–1184, 2013 23662923 10.1111/jeb.12131

[114] S. A. West, A. S. Griffin, A. Gardner, and S. P. Diggle, “Social evolution theory for microorganisms,” *Nature Reviews. Microbiology*, vol. 4, no. 8, pp. 597–607, 2006 16845430 10.1038/nrmicro1461

[115] R. Sanjuán, “The social life of viruses,” *Annu Rev Virol*, vol. 8, no. 1, pp. 183–199, 2021 34242062 10.1146/annurev-virology-091919-071712

[116] S. Martín, and S. F. Elena, “Application of game theory to the interaction between plant viruses during mixed infections,” *The Journal of General Virology*, vol. 90, no. 11, pp. 2815–2820, 2009 19587130 10.1099/vir.0.012351-0

[117] W. M. Wintermantel, A. A. Cortez, A. G. Anchieta, A. Gulati-Sakhuja, and L. L. Hladky, “Co-infection by two criniviruses alters accumulation of each virus in a host-specific manner and influences efficiency of virus transmission,” *Phytopathology*, vol. 98, no. 12, pp. 1340–1345, 2008 19000010 10.1094/PHYTO-98-12-1340

[118] S. A. Felt, Y. Sun, A. Jozwik, A. Paras, M. S. Habibi, D. Nickle, L. Anderson, E. Achouri, K. A. Feemster, A. M. Cárdenas, K. N. Turi, M. Chang, T. V. Hartert, S. Sengupta, C. Chiu, and C. B. López, “Detection of respiratory syncytial virus defective genomes in nasal secretions is associated with distinct clinical outcomes,” *Nature Microbiology*, vol. 6, no. 5, pp. 672–681, 2021 10.1038/s41564-021-00882-3 PMC9098209 33795879

[119] J. Vasilijevic, N. Zamarreño, J. C. Oliveros, A. Rodriguez-Frandsen, G. Gómez, G. Rodriguez, M. Pérez-Ruiz, S. Rey, I. Barba, F. Pozo, I. Casas, A. Nieto, and A. Falcón, “Reduced accumulation of defective viral genomes contributes to severe outcome in influenza virus infected patients,” *PLoS Pathogens*, vol. 13, no. 10, article e1006650, 2017 10.1371/journal.ppat.1006650 PMC5638565 29023600

